# Endothelial Glycocalyx Shedding and Hemodynamic Variables During Hepatic and Pancreatic Resection Surgery

**DOI:** 10.3390/medicina61111938

**Published:** 2025-10-29

**Authors:** Foteini Kavezou, Eleftheria Soulioti, Emmanouil I. Kapetanakis, Evangelos Felekouras, Nikolaos Arkadopoulos, Tzortzis Nomikos, Antonis Galanos, Paraskevi Matsota, Georgia Kostopanagiotou, Tatiana Sidiropoulou

**Affiliations:** 1 Second Department of Anesthesiology, Attikon University Hospital, National and Kapodistrian University of Athens, 12462 Athens, Greece; feniakavezou@gmail.com (F.K.); eleftheriasoulioti@ymail.com (E.S.); matsota@yahoo.gr (P.M.); georgiakostopan@gmail.com (G.K.); 2 Department of Thoracic Surgery, Attikon University Hospital, National and Kapodistrian University of Athens, 12462 Athens, Greece; 3 First Department of Surgery, Laikon General Hospital, National and Kapodistrian University of Athens, 11527 Athens, Greece; evangelosf@hotmail.com; 4 Fourth Department of Surgery, Attikon University Hospital, National and Kapodistrian University of Athens, 12462 Athens, Greece; narkado@hotmail.com; 5 Department of Nutrition and Dietetics, Harokopio University, 17676 Athens, Greece; tnomikos@hua.gr; 6 Laboratory for Research of the Musculoskeletal System (LRMS), KAT General Hospital, School of Medicine, National andKapodistrian University of Athens, 14561 Athens, Greece; galanostat@yahoo.gr

**Keywords:** endothelial glycocalyx, syndecan-1, heparan sulfate, central venous pressure, ischemia/reperfusion, hepatectomy, pancreatectomy

## Abstract

*Background and Objectives*: The endothelial glycocalyx (EG) maintains vascular barrier and homeostasis, but is vulnerable to perioperative stress and ischemia/reperfusion. We evaluated whether central venous pressure (CVP) strategy—low (LCVP, <5 mmHg) versus normal (NCVP, 5–12 mmHg)—and hepatic ischemia/reperfusion during hepatectomy influence perioperative EG shedding in hepatic or pancreatic resections. *Materials and Methods*: A total of 37 adults, out of 40 screened, (18–80 years) scheduled for elective hepatic or pancreatic resection under propofol–remifentanil anesthesia with invasive hemodynamic monitoring, were allocated by initial CVP to LCVP or NCVP protocols and further stratified by ischemia versus no ischemia. Plasma syndecan-1 and heparan sulfate were quantified by ELISA at predefined timepoints (baseline after induction; intraoperative and 2 h post-op). Statistical analyses included nonparametric tests, Friedman with Bonferroni, and ANCOVA adjusted for baseline; *p* < 0.05 significant. *Results*: Thirty-six patients completed analysis (NCVP *n* = 23; LCVP *n* = 13). In procedures without ischemia (*n* = 24; NCVP 16, LCVP 8), heparan sulfate increased over time in both groups; between-group differences in absolute/percentage change were not significant. Syndecan-1 was similar between groups except at 2 h post-op (T3), where LCVP was higher than NCVP (median 9 [11.5] vs. 1.4 [4.5]; *p* = 0.027). In procedures with ischemia (*n* = 12; NCVP 7, LCVP 5), neither biomarker differed between CVP groups at any timepoint. A weak negative CVP–stroke volume variation (SVV) correlation was seen at one timepoint (T1: r = −0.363; *p* = 0.030). *Conclusions*: Major hepatic/pancreatic surgery is associated with measurable EG shedding. Overall, shedding appeared largely independent of CVP strategy and ischemia/reperfusion status, with a late postoperative rise in syndecan-1 under LCVP in non-ischemia cases suggesting potential endothelial cost of aggressive fluid restriction/vasopressor use. These findings highlight the need to refine hemodynamic targets that balance minimizing bleeding with preserving endothelial integrity and suggest that perioperative fluid and vasopressor management may directly influence glycocalyx preservation.

## 1. Introduction

The endothelial glycocalyx (EG) is a dynamic mesh of membrane-bound proteoglycans, glycoproteins, and glycosaminoglycans covering the luminal side of the endothelium [1,2,3]. Its thickness ranges from 20 nm to 4.5 μm, reflecting a constant equilibrium with circulating blood [2,4]. This structure serves crucial physiological roles: it acts as a barrier to large and negatively charged molecules, protects against shear stress, and mediates cell signaling, adhesion, coagulation, and inflammation [3,4,5,6]. Damage to the EG disrupts these functions, leading to capillary leakage, edema, dysregulated inflammation, and impaired vascular homeostasis.

Growing research links glycocalyx degradation to conditions involving ischemia-reperfusion, such as cardiac surgery, sepsis, hypoxia, diabetes, atherosclerosis and viral infections [6,7]. Damage can be assessed histologically or via biomarkers, specifically, elevated blood levels of heparan sulfate (normal value 559 μg/dL) and syndecan-1 (normal value 1.7 μg/dL) [7,8,9,10].

Hepatic ischemia-reperfusion also directly affects liver sinusoidal endothelial cells (LSECs), which are among the first to be injured, showing swelling, blebbing, and detachment from the sinusoidal wall, thereby compromising perfusion and amplifying reperfusion injury [11,12]. Nevertheless, surviving LSECs secrete angiocrine factors such as hepatocyte growth factor (HGF), Wingless-type MMTV integration site family member 2 (Wnt2), and angiopoietin-2, driving hepatocyte proliferation, angiogenesis, and coupling vascular repair with parenchymal regeneration [13,14].

Hepatectomy, a procedure involving ischemia-reperfusion, offers a relevant model to study EG injury. Techniques such as the Pringle maneuver or hepatic vascular occlusion reduce blood loss by limiting hepatic blood flow [15,16,17]. Anesthesia approaches, particularly low Central Venous Pressure (CVP) (CVP ≤ 5 mmHg), further reduce bleeding by minimizing hepatic venous congestion [15].

The purpose of this study is to investigate the possible damage of the EG in patients undergoing hepatectomy, with or without ischemia, or pancreatectomy, depending on the anesthesia technique used (low versus normal CVP) and to highlight the technique that is most beneficial for its preservation. We hypothesized that a lower CVP would result in reduced glycocalyx shedding compared with a normal CVP strategy, and that ischemia/reperfusion would exacerbate glycocalyx injury.

## 2. Materials and Methods

### 2.1. Participants and Eligibility Criteria

The trial was conducted at the “Attikon University Hospital” in Athens, Greece. The trial was approved by the Institutional Scientific Review Board (no 1481/22-04-2016). Written informed consent was obtained from all the participants the day before surgery. The trial followed the principles of the revised Declaration of Helsinki [18]. We included adult patients (age 18 to 80 years), without or with mild liver disease (Child-Pugh classification A) planned to undergo an elective hepatic or pancreatic resection under general anesthesia, for malignant tumors (hepatocellular carcinoma, cholangiocarcinoma, pancreatic adenocarcinoma) and metastatic disease, as well as selected benign lesions. Exclusion criteria included patients with allergy or contraindication to the administered drugs, patients with uncontrolled diabetes, severe kidney disease, heart failure, severe valvular disease, pulmonary hypertension, severe chronic obstructive pulmonary disease or severe liver disease (Child-Pugh classification B or C). The presence of liver disease was evaluated as a possible risk factor for EG damage.

### 2.2. Perioperative Management

Prior to anesthesia induction, besides the standard operating room monitoring, depth of anesthesia monitoring was also used and a radial artery catheter was inserted for continuous monitoring of blood pressure, with a Flotrack transducer (Edwards Lifesciences, Irvine, CA, USA), which was connected both to the operating room monitor and to a Vigileo monitor (Edwards Lifesciences, Irvine, CA, USA). The arterial waveform was analyzed by the Vigileo monitor to obtain more hemodynamic parameters such as Cardiac Output (CO), Cardiac Index (CI), Stroke Volume (SV) and Stroke Volume Variation (SVV). If blood coagulation values were normal, either a thoracic epidural or a single shot spinal were performed, for adequate analgesia perioperatively. Induction of anesthesia was accomplished with propofol, fentanyl and rocuronium. After tracheal intubation, mechanical positive pressure ventilation was applied and maintenance of anesthesia was obtained with continuous intravenous infusion of propofol and remifentanil, with bispectral index values targeted at 40–60. After anesthesia induction a central venous catheter was placed and connected to the operating room monitor for CVP monitoring.

### 2.3. Perioperative Fluid Regimen Protocol

**Figure 1:**
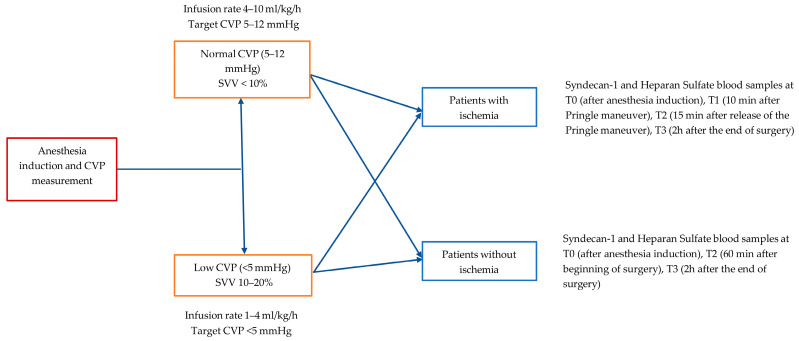
Schematic representation of the protocol.

A schematic representation of the protocol is shown in Figure 1. Patients were assigned to two groups, based on the first central venous pressure measurement, which was measured immediately after anesthesia induction, before fluid administration or vasoactive drugs: a group with low initial CVP value (<5 mmHg) (group LCVP) and a group with normal CVP (5–12 mmHg) (group NCVP). The perioperative fluidprotocol was the same for the two groups. The LCVP group received balanced crystalloids with a rate of 1–4 mL/kg/h to maintain a low CVP (<5 mmHg). The NCVP group received crystalloids with a rate of 4–10 mL/kg/h to maintain a normal CVP value (5–12 mmHg). A prerequisite in both groups was the maintenance of satisfactory diuresis (>0.5 mL/kg/h) and hemodynamic stability, that was maintained with vasoactive drugs (ephedrine, phenylephrine) whenever needed. Blood and blood products were given, targeting to Hb 7–10 g/dL and an INR < 1.5.

Patients were also classified based on the surgical procedure they underwent, if there was a Pringle maneuver involving global hepatic ischemia (patients with ischemia) or if there was a selective hepatic vascular exclusion (patients without ischemia). The decision regarding Pringle maneuver versus selective vascular exclusion was at the discretion of the operating surgeon based on intraoperative considerations. During surgery an investigator was present to take notes, concerning ischemia time, amount of fluids or blood products infused, anesthesia drugs or inotropes and vasopressors given, blood loss, and other adverse events.

### 2.4. Assessment of Endothelial Glycocalyx Disruption

To determine blood levels of heparan sulfate and syndecan-1, blood samples were collected at these timepoints:

- Patients with ischemia: T0 (after anesthesia induction), T1 (10 min after Pringle maneuver), T2 (15 min after release of the Pringle maneuver), T3 (2 h after the end of surgery)
- Patients without ischemia: T0 (after anesthesia induction), T2 (60 min after the beginning of surgery), T3 (2 h after the end of surgery)

Blood samples were analyzed in a laboratory, with commercially available ELISA kits (Human Syndecan-1 DuoSet ELISA 15 plates, BIOTECHNE, R&D SYSTEMS, Minneapolis, MN, USA, catalogue number DY2780 and Human heparan sulfate, HS ELISA Kit, CUSABIO, Houston, TX, USA, catalogue number CSB-E09585h) for the quantitative determination of heparan sulfate and syndecan-1 blood concentration. All samples were analysed in duplicate for internal validity.

### 2.5. Statistical Analysis

As the study was exploratory in nature, no formal power calculation was performed prior to study initiation. Also, it was not originally powered to detect small between-group differences but to generate preliminary data on endothelial glycocalyx dynamics in the perioperative setting.

Continuous variables are described using the mean and standard deviation or median and interquartile range (if data did not follow a normal distribution) and categorical variables using frequencies (*n*) and percentages (%). The Shapiro-Wilks test was used to test for normal distribution of data. Comparison for homogeneity between normal (NCVP) and low (LCVP) CVP group with respect to demographic and clinical indicators was performed using the independent samples *t*-test, the Chi-square test, and Fisher’s exact test. The Mann-Whitney test and the median test were for the comparison between the two groups at each time assessment, while the longitudinal comparison of variables for each group separately was done using the Friedman test and the Bonferroni correction for all pairwise comparisons. Sensitivity analysis of the variables regarding the homogeneity of the groups at time T0 (baseline-balance) was performed using two methods: (1) The percentage change from baseline at all time assessments where we compare these percentage changes between the two groups with the Mann-Whitney test and the median test because the data do not have a normal distribution and (2) The absolute change from baseline at all time points where we compare the absolute change in the variables (dependent variable) between the two groups (factor) and the estimate of the variables at time T0 (covariate) using the analysis of covariance (ANCOVA) model. All statistical analyses were performed with the statistical package SPSS vr 21.00 (IBM Corporation, Somers, NY, USA). All tests are two-sided. A *p*-value < 0.05 was defined as a statistically significant level.

## 3. Results

**Figure 2:**
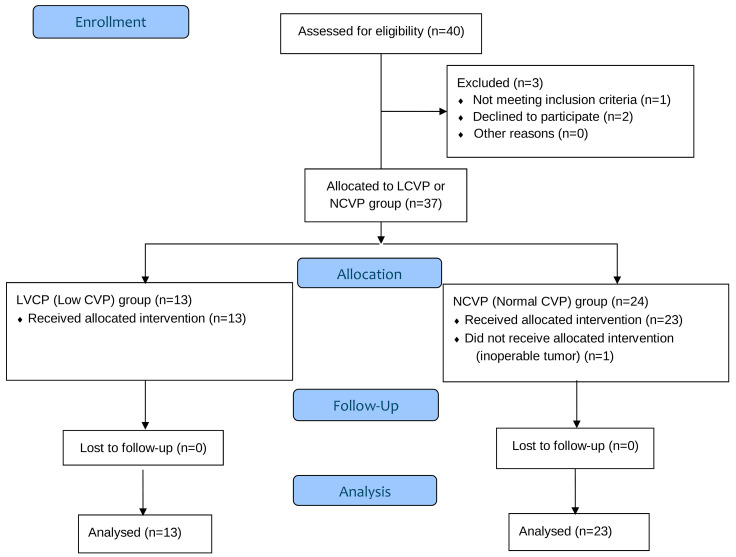
Consort flow diagram of the study.

Forty surgical patients were assessed for enrollment in the study. Two patients declined to participate and one didn’t meet the inclusion criteria. A total of 37 patients were allocated to either LCVP group or NCVP group, as shown in Figure 2. One patient from the NCVP group didn’t receive the allocated intervention and was excluded from the data analysis due to an inoperable tumor detected in the early stages of the procedure. Therefore, the study protocol was applied to 36 patients without complications. No statistically significant differences were observed between the groups regarding demographic and clinical characteristics, concomitant diseases or intraoperative data, as seen in Table 1 and Table 2.

**Figure 3:**
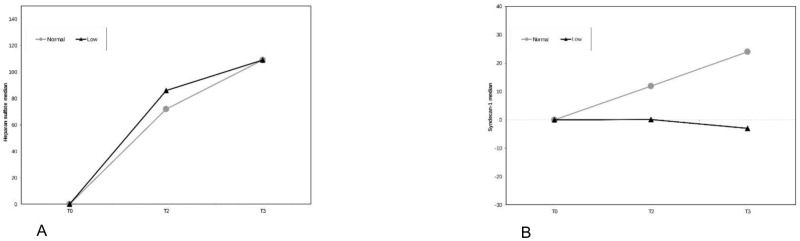
Percentage change from time T0 of heparan sulfate (**A**) and syndecan-1 (**B**) between the two groups in patients without ischemia.

Due to the different collection of blood samples for heparan sulfate and syndecan-1, data were analyzed separately for patients with or without ischemia. Both absolute and relative changes from baseline (T0) were analysed. Because baseline biomarker values varied between individuals, presenting results as a percentage change allowed for clearer assessment of intra-patient trajectories while controlling for inter-individual variability. Regarding procedures without ischemia/reperfusion, which included a total of 24 patients, 16 in the NCVP group and 8 in the LCVP group, there was no significant difference in the absolute change from time T0 to times T2 (*p* = 0.443) and T3 (*p* = 0.244) of the heparan sulfate variable adjusted for the value at time T0 between the two groups or the percentage change from time T0 to times T2 (*p* = 0.787) and T3 (*p* = 1.000) (Figure 3A). Heparan sulfate values increased over time significantly in both groups (Table 3). Concerning syndecan-1, there was no significant difference in the absolute change from time T0 to times T2(*p* = 0.742) and T3 (*p* = 0.808) adjusted for the value at time T0 between the groups, or the percentage change from time T0 to times T2 (*p* = 1.000) and T3 (*p* = 0.742) between the groups (Figure 3B). A significant difference was detected at T3 between normal and low CVP groups (1.4 [4.5] vs. 9 [11.5], *p* = 0.027) (Table 3).

**Figure 4:**
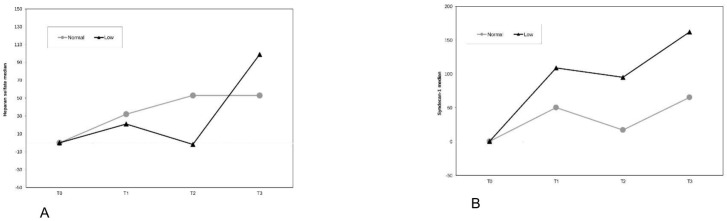
Percentage change from time T0 of heparan sulfate (**A**) and syndecan-1 (**B**) between the two groups in patients with ischemia.

Regarding procedures with ischemia/reperfusion, which included 12 patients,7 in the NCVP group and 5 in the LCVP group, there was no significant difference in the absolute change of heparan sulfate values from time T0 to times T1 (*p* = 0.425), T2 (*p* = 0.243), T3 (*p* = 0.882) of the heparan sulfate variable adjusted for the value at time T0 or the percentage change from time T0 to times T1 (*p* = 0.639), T2 (*p* = 0.432), T2 (*p* = 0.876) of the between the groups (Figure 4A). Accordingly, there was no significant difference in the absolute change from time T0 to times T1 (*p* = 0.609), T2 (*p* = 0.585), T3 (*p* = 0.148) of the syndecan-1 variable adjusted for the value at time T0 between the groups or the percentage change from time T0 to times T1 (*p* = 0.530), T2 (*p* = 0.432), T2 (*p* = 0.429) of the syndecan-1 variable between the groups (Figure 4Β). Heparan sulfate and syndecan-1 values over time can be seen in Table 4.

We also investigated a possible correlation between CVP and SVV. There is a low negative statistically significant correlation at time T1 (r = −0.363, *p* = 0.030), as seen in Table 5.

## 4. Discussion

This observational prospective cohort evaluated perioperative hemodynamic variables and endothelial glycocalyx shedding during hepatic and pancreatic resections, with or without ischemia-reperfusion, under different central venous pressure management strategies. Circulating biomarkers of glycocalyx degradation (syndecan-1 and heparan sulfate) increased across all groups, suggesting that major abdominal surgery itself significantly compromises endothelial integrity. CVP strategy and ischemia-reperfusion status did not consistently alter the extent of glycocalyx shedding, except for a late postoperative rise in syndecan-1 observed in the low-CVP group without ischemia.

The late postoperative rise in syndecan-1 in the low-CVP cohort without ischemia suggests that the combination of stringent fluid restriction and compensatory vasopressor use may stress the endothelium and degrade the glycocalyx, even when overt ischemia-reperfusion is absent. Low-CVP strategies reduce blood loss and hepatic congestion, but relative hypovolemia can increase vasopressor requirements; depending on dose and context, catecholamines may maintain mean arterial pressure yet still impair microvascular coherence and promote endothelial injury, potentially amplifying glycocalyx shedding and capillary leak. Clinically, this pattern could translate into tissue edema, subtle organ hypoperfusion, and slower recovery, arguing for individualized targets that temper fluid restriction, avoid excessive vasopressor exposure, and incorporate perfusion-oriented monitoring (e.g., microcirculatory indices or perioperative glycocalyx biomarkers). Future trials comparing low-CVP versus goal-directed strategies with standardized vasopressor protocols and serial syndecan-1/heparan-sulfate measurements are warranted [19,20,21,22,23,24,25,26,27].

Our findings align with other studies showing that major abdominal surgery is associated with significant endothelial activation and glycocalyx disruption, as reflected by increased plasma syndecan-1 and heparan sulfate levels [22,23]. The perioperative inflammatory and hemodynamic stressors—including surgical trauma, anesthetic agents, fluid shifts, and blood loss—contribute to enzymatic cleavage of glycocalyx components [1,6,7,28]. Gregersen et al. demonstrated that syndecan-1 levels rise markedly during open abdominal surgery, and these increases are greater in patients with severe postoperative morbidity [23]. This suggests that EG injury may be both a marker and a mediator of adverse outcomes.

In our study, the choice of CVP strategy did not yield consistent differences in glycocalyx biomarkers, except for a significantly higher syndecan-1 level at T3 in the LCVP group without ischemia. Although low CVP is well-established for reducing blood loss and improving the surgical field during hepatectomy, it may also lead to relative hypovolemia, necessitating vasopressor use to maintain arterial pressure [3,4,15,16,17,29]. Vasopressors, while effective in sustaining perfusion pressure, may increase endothelial shear stress or microvascular resistance, potentially contributing to glycocalyx injury. Similar observations have been made in other perioperative contexts, where aggressive fluid restriction was linked to endothelial perturbation despite reduced bleeding risk [6].

Dynamic preload indices such as stroke volume variation (SVV) have demonstrated clear advantages over static measures like central venous pressure (CVP) in guiding intraoperative fluid therapy. Unlike CVP, which poorly reflects intravascular volume status and fails to predict fluid responsiveness, SVV provides a real-time, physiology-based assessment of preload dependency under controlled ventilation conditions. Several meta-analyses and randomized trials have confirmed that SVV-guided goal-directed fluid management results in more accurate fluid optimization, reduced intraoperative fluid administration, and improved hemodynamic stability compared with conventional CVP-guided strategies. In high-risk abdominal and hepatic surgery, SVV-targeted protocols have been associated with fewer hypotensive episodes, lower postoperative lactate levels, and shorter hospital stays, suggesting enhanced tissue perfusion and reduced fluid overload. Therefore, integrating SVV into perioperative hemodynamic management offers a more individualized and responsive approach to maintaining circulatory stability than relying on static CVP values alone [30,31,32,33].

In hepatobiliary surgery, the endothelial glycocalyx is particularly vulnerable to ischemia–reperfusion and surgical stress. In liver transplantation, plasma syndecan-1 rises sharply after reperfusion and has been associated with postoperative acute kidney injury [34,35]. Human graft-level studies confirm washout of syndecan-1 during reperfusion [35], and more recent machine-perfusion data suggest that glycocalyx degradation products measured during hypothermic oxygenated perfusion may predict early allograft dysfunction [36]. In pancreatic surgery, clinical evidence is emerging: an ongoing randomized study in pancreatectomy is directly measuring perioperative syndecan-1 and heparan sulfate under goal-directed restrictive therapy, comparing albumin with gelofusine as colloid carriers [37]. More broadly, major open abdominal procedures have demonstrated perioperative increases in circulating glycocalyx-shedding biomarkers [23], and a large randomized trial in high-risk abdominal surgery found that prophylactic norepinephrine at induction reduced hypotension and complications compared with ephedrine, supporting a fluid-sparing, vasopressor-supported approach that is plausibly protective of the glycocalyx [38]. In addition, sublingual measurement of the perfused boundary region (PBR) provides a feasible intraoperative tool for bedside glycocalyx monitoring and has been linked to systemic glycocalyx integrity [39]. Taken together, these data suggest that during liver and pancreatic surgery, the EG is highly susceptible to injury, and that careful hemodynamic management—prioritizing vasopressor-guided pressure support over liberal fluid loading—may help mitigate glycocalyx degradation.

Dynamic preload indices such as SVV may offer a more precise guide to fluid therapy than static CVP measurements [40,41,42]. In the randomized trial by Hsieh et al., stroke volume variation (SVV)–guided fluid therapy during hepatectomy was associated with measurable differences in postoperative liver injury markers [43]. Although overall complication rates did not differ between low-SVV (≤10%) and high-SVV (>10%) groups, patients managed with a high-SVV strategy exhibited significantly higher postoperative alanine aminotransferase (ALT) levels, suggesting greater hepatocellular injury. This finding implies that restrictive fluid administration leading to higher SVV may compromise hepatic microcirculation or exacerbate ischemia–reperfusion stress despite maintenance of adequate mean arterial pressure. The authors noted that ALT elevation, a recognized marker of hepatocellular damage and predictor of postoperative morbidity and mortality, likely reflected transient hepatic injury related to perioperative hypoperfusion. Thus, while SVV-guided restriction did not increase overt complications, higher SVV values were associated with biochemical evidence of postoperative liver injury, underscoring the importance of balancing fluid optimization and hepatic perfusion during liver resection. No such correlation was observed in our study.

Similarly, the study by Saito et al. demonstrated a clear relationship between stroke volume variation (SVV), intraoperative fluid balance, and postoperative outcomes in liver resection [44]. Specifically, higher SVV values—reflecting a more restrictive intraoperative fluid strategy—were negatively correlated with intraoperative blood loss (IBL) and were associated with fewer postoperative complications, fewer transfusions, and shorter hospital stays compared with low-SVV management. Maintaining high SVV requires limiting fluid administration, which reduces hepatic venous congestion and bleeding from the resection surface without causing lasting renal impairment, as transient postoperative creatinine increases normalized by discharge. Conversely, low SVV, indicating higher intravascular volume, was linked to increased blood loss, transfusion need, and complications such as ascites. Overall, these findings highlight that SVV-guided restrictive fluid management can optimize intraoperative hemodynamics, minimize blood loss, and improve postoperative recovery in hepatectomy. No such correlation was observed in our study. In contrast, more blood transfusions were given in pancreatectomies and patients with high SVV values.

In our study, we found a low negative statistically significant correlation only at one timepoint, while prior randomized trials have shown that SVV-guided fluid management can reduce intraoperative fluid administration without compromising hemodynamic stability [18,20]. Nevertheless, this hemodynamic optimization does not translate into detectable differences in glycocalyx shedding.

Hepatic ischemia-reperfusion is known to trigger oxidative stress, inflammation, and activation of sheddases such as matrix metalloproteinases, leading to rapid glycocalyx degradation [5,6,7,16]. Experimental work by van Golen et al. and clinical studies have demonstrated marked increases in syndecan-1 and heparan sulfate within minutes of reperfusion [5,9,16,21]. In our ischemia subgroup, however, these increases were not significantly different between the two groups. This may be due to the small sample size or to the fact that the ischemia-reperfusion insult overshadowed any subtle effects of hemodynamic strategy.

Interestingly, recent work by Weinberg et al. described distinct patterns of biomarker release following major surgery, with the magnitude of syndecan-1 and heparan sulfate increase depending on surgical duration, blood loss, and procedure type [14]. This variability may explain why our biomarker trajectories did not differ significantly between subgroups.

This study has several limitations. First, the relatively small sample size, particularly in the ischemia subgroup, may limit statistical power. As stated before, no formal power calculation was performed prior to study initiation because this was an exploratory study. Post-hoc power analysis was performed for the primary outcome (syndecan-1 changes). The achieved power was 12% for detecting the observed difference at T3 in the non-ischemia subgroup. A larger, well-controlled study would give more definite results.

Second, although anesthesia and fluid management were standardized, differences in surgical complexity could have influenced the extent of glycocalyx injury. The choice of surgical technique, as well as the decision to apply the Pringle maneuver or selective vascular occlusion, was at the discretion of the operating surgeon, according to the specific requirements of each case. Therefore, a uniform surgical protocol was not applied, which represents another factor potentially affecting the results. Nonetheless, these intraoperative decisions were made with the aim of addressing each case’s complexity and ensuring optimal patient care

Third, syndecan-1 and heparan sulfate are the most widely used circulating biomarkers of endothelial glycocalyx degradation and have been validated in both experimental and clinical settings. However, they are not highly specific, as elevated levels may also reflect general endothelial activation and systemic inflammation. Other biomarkers under investigation include hyaluronan, chondroitin sulfate, and soluble thrombomodulin. Biomarker measurements offer only indirect evidence of glycocalyx disruption; integrating these with imaging approaches (e.g., sublingual microvascular glycocalyx thickness via sidestream dark field microscopy) or functional endothelial assessments could provide more comprehensive insights.

Preservation of the endothelial glycocalyx is increasingly recognized as a therapeutic target in perioperative care. Although our findings do not demonstrate a clear advantage of one CVP strategy over another with respect to glycocalyx preservation, they highlight the importance of avoiding both extreme hypovolemia and excessive vasopressor use. An individualized fluid management approach—incorporating stroke volume variation (SVV), cardiac output (CO) monitoring, and early detection of endothelial stress—may help optimize the balance between hemostasis, organ perfusion, and vascular protection.

## 5. Conclusions

Both hepatic and pancreatic resections were associated with measurable perioperative glycocalyx shedding, largely independent of ischemia-reperfusion or CVP strategy at most timepoints. The late postoperative rise in syndecan-1 observed in the low-CVP group without ischemia highlights the complex interplay between fluid restriction, vasopressor use, and endothelial integrity. However, interpretation of these findings is limited by the small sample size and the observational study design, which preclude firm causal conclusions. Future research should include larger, randomized controlled trials and, where feasible, direct imaging or microcirculatory assessment of the glycocalyx to better elucidate perioperative endothelial responses and guide protective anesthetic and surgical strategies.

## Figures and Tables

**Table 1:** Demographic characteristics of all groups.

	Normal CVP Group Without Ischemia (*n* = 16)	Low CVP Group Without Ischemia (*n* = 8)	*p*	Normal CVP Group with Ischemia (*n* = 7)	Low CVP Group with Ischemia (*n* = 5)	*p*
Age (years)	61.8 ± 13.0	63.1 ± 11.9	0.814	58.7 ± 13.1	58.2 ± 9.8	0.943
M/F	7/9	6/2	0.211	4/3	3/2	1.000
BMI (kg/m^2^)	26.8 ± 2.2	24.8 ± 5.1	0.196	27.0 ± 4.1	26.9 ± 1.4	0.956
ASA (II/III)	5/10/1	4/4/0	0.565	1/5/ 1	2/3/0	0.462
Smoking (n/y)	12/4	6/2	0.565	5/2	4/1	1.000
Presence of Concomitant disease (n/y)						
Peripheral vascular disease	15/1	8/0	1.000	6/1	5/0	1.000
Arrhythmias	14/2	7/1	1.000	7/0	5/0	1.000
Dyslipidemia	14/2	7/1	1.000	5/2	4/1	1.000
Hypertension	11/5	5/3	1.000	6/1	3/2	1.000
Diabetes	14/2	6/2	1.000	6/1	5/0	1.000
Hypothyroidism	14/2	7/1	1.000	5/2	4/1	1.000
Renal failure	15/1	8/0	1.000	6/1	5/0	1.000
Metastatic cancer	14/2	6/2	0.578	5/2	1/4	0.242
Solid tumor no metastasis	4/12	2/6	1.000	2/5	4/1	0.242

Data are presented as mean ± SD, median [interquartile range] or number (percentage within group). Abbreviations: CVP: Central Venous Pressure; M/F: Male/Female; BMI: Body Mass Index; ASA: American Society of Anesthesiologists (ASA) Physical Status Classification System; n/y: no/yes.

**Table 2:** Intraoperative data.

	Normal CVP Group Without Ischemia (*n* = 16)	Low CVP Group Without Ischemia (*n* = 8)	*p*	Normal CVP Group with Ischemia (*n* = 7)	Normal CVP Group with Ischemia (*n* = 5)	*p*
Procedure (hepatectomy/pancreatectomy)	8/8	4/4	1.000	6/1	4/1	1.000
Anesthesia duration (min)	452 (130–660)	480 (220–640)	0.624	235 (180–660)	360 (175–515)	0.755
Procedure duration (min)	392 (105–590)	350 (180–600)	0.671	180 (120–600)	290 (120–420)	0.530
Ischemia time (min) (pringle maneuver)	-	-	-	20 (13–28)	35 (13–50)	0.149
Crystalloids (mL)	3350 (900–6500)	3500 (1200–4600)	0.671	2000 (1700–4700)	3700 (1300–5700)	0.755
Phenylephrine (mg)	0.6 (0–3)	0.4 (0–1.5)	0.720	0.6 (0–2.7)	0.8 (0.3–1.5)	0.639
Ephedrine (mg)	2.5 ± 6.5	3.7 ± 4.8	0.413	0.7 ± 1.8	0.0 ± 0.0	0.755

Data are presented as mean ± SD, median [min-max] or number (percentage within group). Abbreviations: CVP: Central Venous Pressure.

**Table 3:** Heparan Sulfate and Syndecan-1 laboratory values in patients without ischemia/reperfusion.

	Τ0	Τ1	Τ3	*p*-Value Within Groups
**Heparan Sulfate** (ng/mL)				
NCVP (*n* = 16)	258.7 [137.5]	427.8 [361.1]	538.6 [340.1]	0.007
LCVP (*n* = 8)	305.4 [100.3]	654.5 [643.4]	644.3 [873.8]	0.005
***p*** **-value (between groups)**	0.667	0.193	0.667	
**Syndecan-1** (ng/mL)				
NCVP (*n* = 16)	1.1 [7.9]	1.5 [16.3]	1.4 [14.4]	0.185
LCVP (*n* = 8)	7.3 [17.8]	7.3 [14.2]	9.0 [11.5]	1.000
***p*** **-value (between groups)**	0.193	0.193	0.027	

Data are presented as median [interquartile range]. Abbreviations: NCVP: Normal Central Venous Pressure group; LCVP: Low Central Venous Pressure group.

**Table 4:** Heparan Sulfate and Syndecan-1 laboratory values in patients with ischemia/reperfusion.

	Τ0	Τ1	Τ2	Τ3	*p*-Value Within Groups
**Heparan Sulfate** (ng/mL)					
NCVP (*n* = 7)	254.3 [131.7]	381.2 [242.9]	388.8 [290]	309.8 [383.4]	0.419
LCVP (*n* = 5)	272.7 [289.8]	281.9 [371.4]	250.1 [396.4]	273.8 [670.7]	0.668
***p*** **-value (between groups)**	NS	NS	0.242	0.567	
**Syndecan-1** (ng/mL)					
NCVP (*n* = 7)	1.2 [1.9]	2.4 [2.2]	1.7 [1.8]	3.2 [4.6]	0.086
LCVP (*n* = 5)	1.1 [34.7]	5.6 [42.3]	6.1 [37.3]	7.2 [38.6]	0.062
***p*** **-value (between groups)**	1.000	1.000	0.242	0.567	

Data are presented as median [interquartile range]. Abbreviations: NCVP: Normal Central Venous Pressure group; LCVP: Low Central Venous Pressure group.

**Table 5:** Correlation between CVP and SVV indices.

	CVP1	CVP2	CVP3	CVP4
SVV1	r = −0.363			
SVV2		r = −0.307		
SVV3			r = 0.014	
SVV4				r = −0.043

Abbreviations: CVP: Central Venous Pressure; SVV: Stroke Volume Variation.

## Data Availability

The data presented in this study are available on request from the corresponding author.

## Abbreviations

The following abbreviations are used in this manuscript:

EG	Endothelial Glycocalyx
LSECs	Liver sinusoidal endothelial cells
HGF	Hepatocyte growth factor
Wnt2	Wingless-type MMTV integration site family member 2
CVP	Central Venous Pressure
CO	Cardiac Output
CI	Cardiac Index
SV	Stroke Volume
SVV	Stroke Volume Variation
LCVP	Low Central Venous Pressure
NCVP	Normal Central Venous Pressure
BMI	Body Mass Index
ASA	American Society of Anesthesiologists (ASA) Physical Status Classification System
PBR	Perfused boundary region
ALT	Alanine aminotransferase
IBL	Intraoperative blood loss
